# Novel reconstruction using pedicled ileocolic interposition after laparoscopic total gastrectomy: A report of two cases

**DOI:** 10.1016/j.ijscr.2024.110501

**Published:** 2024-10-28

**Authors:** Takeshi Ono, Yuichiro Hirata, Koji Kato, Masashi Nagata, Satoru Higa, Hisashi Fujiwara

**Affiliations:** a Department of Surgery, Okinawa Kyodo Hospital, Okinawa, Japan; b Department of Gastrointestinal Surgery, Tokyo Medical and Dental University, Tokyo, Japan

**Keywords:** Ileocolic reconstruction, Laparoscopic surgery, Total gastrectomy, Gastric cancer

## Abstract

**Introduction:**

Although Roux-en-Y reconstruction using the jejunum is generally performed after laparoscopic total gastrectomy, the postoperative function is inadequate. We designed a novel reconstruction technique using pedicled ileocolic interposition with laparoscopic anastomosis of the esophagus and ileum, and further anastomosis of the colon and duodenum. Two patients were treated with this technique.

**Case presentation:**

Case 1 involved a 74-year-old man with multiple gastric cancer. Case 2 involved a 77-year-old man with extensive scirrhous esophagogastric junction cancer and esophageal invasion of 2 cm. These 2 patients underwent laparoscopic total gastrectomy and pedicled ileocolic interposition anastomosis. The patients were discharged without major complications.

**Discussion:**

We anticipate that the implementation of this reconstruction method will enhance the quality of life of patients after total gastrectomy, particularly in terms of minimizing esophageal reflux and facilitating oral ingestion. To our knowledge, this is the first report of laparoscopic reconstruction with a pedicled ileocolic interposition after total gastrectomy.

**Conclusion:**

Pedicled ileocolic interposition is characterized by the expectation of good postoperative function owing to the anti-reflux mechanism of the ileocecal valve and adequate reservoir function of the cecum and colon.

## Highlights

• We designed a novel reconstruction technique using pedicled ileocolic interposition with the laparoscopic anastomosis of the esophagus and ileum, and a further anastomosis of the colon and duodenum. Two patients were treated with the technique. The patients were discharged without major complications.
• There have only been a few scattered reports of ileocolic reconstruction for total gastrectomy which were mostly published from the 1930s to the 1950s. However, such studies have almost completely disappeared since around the year 2000. With the development of laparoscopic techniques and improved surgical safety, ileocolic reconstruction has been reconsidered as a reconstruction method.
• We anticipate that the implementation of this reconstruction method will enhance the quality of life for patients after total gastrectomy, particularly in terms of minimizing esophageal reflux and facilitating oral ingestion.

## 1 Introduction

Distal gastrectomy and proximal gastrectomy preserve a certain amount of residual stomach, which allows for a somewhat better postoperative function [1]. In particular, various new reconstructive methods have become popular in recent years for proximal gastrectomy, such as double-tract and the double flap technique [2]. However, after total gastrectomy, Roux-en-Y reconstruction using the small intestine is performed as before, and its postoperative function is not sufficient [3]. Therefore, we designed a new original reconstruction method using a pedicled ileocolic interposition graft after laparoscopic total gastrectomy. In anticipation of good postoperative function due to the anti-reflux mechanism of the ileocecal valve and adequate reservoir function of the cecum and colon, we applied this method in the treatment of two patients. Patients who fully understood and consented to surgery were eligible for inclusion in the study. We have reported this case report in line with the SCARE criteria [4].

## 2 Case presentation

### 2.1 Case 1

The patient was a 74-year-old man with performance status (PS) 1 in whom a Type 1 upper gastric tumor was incidentally found on a computed tomography (CT) scan. An endoscopic examination revealed Type 1 upper gastric tumor and an additional Type0-IIa + IIc cancer in the gastric antrum. Histological examination of the endoscopic biopsy specimens revealed adenocarcinomas in both tumors. The patient was diagnosed with multiple gastric cancers cT2N0 and cT1bN0, and laparoscopic total gastrectomy was planned without neoadjuvant therapy according to the Japanese gastric cancer treatment guidelines.

### 2.2 Case 2

The patient was a 77-year-old man and PS2 with upper abdominal pain. An endoscopic examination revealed a scirrhous gastric cancer that extending from the esophagogastric junction to the gastric angle. A histological examination of the endoscopic biopsy specimen confirmed poorly differentiated adenocarcinoma. Enhanced CT revealed submucosal esophageal invasion exceeding 2 cm with no swollen lymph nodes or peritoneal dissemination nodes. The patient was diagnosed with cT4aN0 gastric cancer, and laparoscopic total gastrectomy, splenectomy, and distal esophagectomy without neoadjuvant therapy were planned according to the Japanese gastric cancer treatment guidelines. No staging laparoscopy was performed.

### 2.3 Surgical procedure

**Fig. 1:**
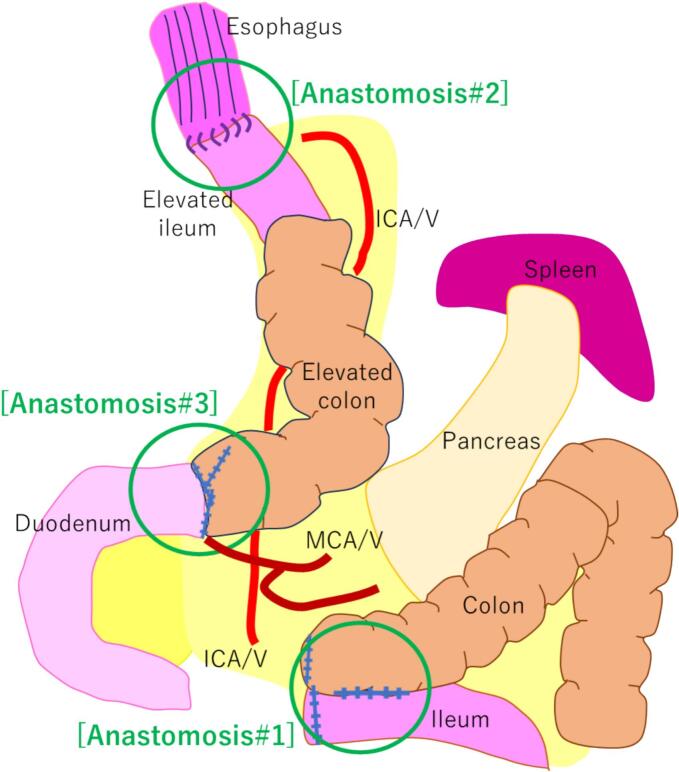
An illustration of the complete reconstruction process. Three sites of anastomosis are indicated by green circles; in sequence, (#1) anastomosis between the ileum and colon under a small laparotomy, (#2) anastomosis between the esophagus and elevated ileum under laparoscopy, (#3) anastomosis between elevated colon and duodenum under laparoscopy. (For interpretation of the references to colour in this figure legend, the reader is referred to the web version of this article.)

**Fig. 2:**
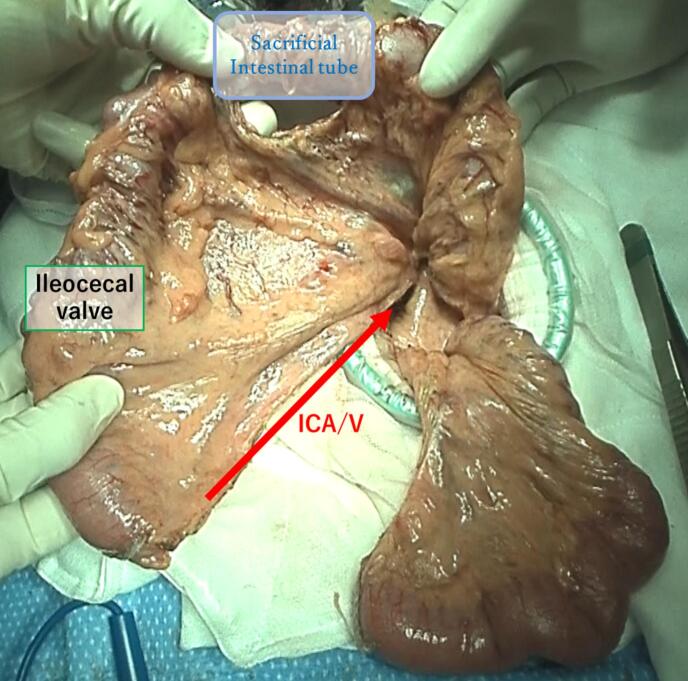
Creation of a pedicled ileocolic graft. The ileum and its mesentery were transected along the ileocecal artery and vein on the oral side of the pedicled graft. We preserved the colonic mesentery and its marginal vessels on the anal side of graft, but instead created 5-cm sacrificial colon.

Total gastrectomy was performed laparoscopically with a common trapezoidal 5-port to complete resection and lymph node dissection. In Patient 2, the thoracic esophageal resection margin was determined to be frozen, and negative. The complete reconstruction process is illustrated in Fig. 1. The scope port wound was enlarged according to the amount of specimen retrieved and a platform was attached for single-site surgery. The right colon used for the reconstruction was mobilized laparoscopically. Moving on to reconstruction, we created a pedicled ileocolic graft (Fig. 2). Approximately 10 cm of the terminal ileum and 10 cm of the ascending colon were used. The ileum was dissected and the mesentery was cut along the ileocecal artery. The hepatic flexure of the colon was resected as a 5-cm sacrificial intestinal tube. Under a small laparotomy, the ileum colon was reconstructed with functional end-to-end anastomosis (anastomosis #1, graft extraction site). Returning to the laparoscopic operation again, esophagus-ileum anastomosis (anastomosis #2) was performed using the overlap method. Finally, we formed a colonic duodenum (anastomosis #3) with delta anastomosis. Presurgical mechanical bowel preparation is necessary to reduce the severity of infectious complications, while assuming the occurrence of anastomotic leakage. All procedures were completed with adequate abdominal cleaning and drain placement.

### 2.4 Pathological diagnosis

Case1: Multiple gastric cancer, adenocarcinoma, pT2 and pT1bN2M0, pStageIIB.

Case2: Adenocarcinoma, pT4aN3bM1(CY+,P1), pStageIV.

### 2.5 Postoperative findings and outcomes

The operation times in the two patients were 363 and 406 min, respectively. Intraoperative blood loss was 200 and 10 mL. Oral intake of liquid was started on postoperative day 2 and they began eating meals on the 7th postoperative day, after contrast studies were performed. Patient 2 had pleural effusion, which was suspected to be pyothorax, and drainage was performed; however, the effusion was sterile. The patients were discharged on the 20th and 21st postoperative days, respectively.

**Fig. 3:**
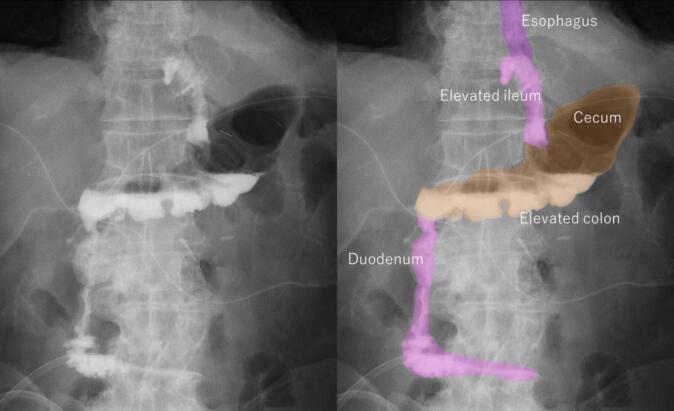
Postoperative fluorography findings at one week after the operation. After stagnating in the colon for approximately 2 min, the contrast agent flowed into the duodenum.

**Fig. 4:**
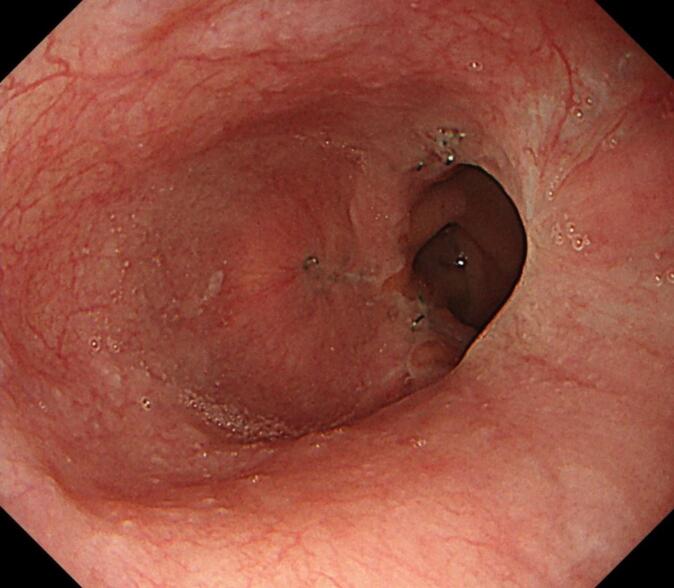
Postoperative endoscopic findings on 3 months after operation. There was no anastomotic stricture or reflux esophagitis.

Both fluorography and endoscopy after the surgery revealed the absence of anastomotic strictures and reflux esophagitis (Fig. 3, Fig. 4). None of the patients reported reflux symptoms. Reconstruction of the lower esophagus and cardia was performed as intended. They are currently undergoing postoperative adjuvant chemotherapy.

## 3 Discussion

To our knowledge, this is the first report of laparoscopic reconstruction with a pedicled ileocolic interposition after total gastrectomy. In fact, there have only been a few scattered reports of ileocolic reconstruction for total gastrectomy which were mostly published in the 1900s [[5], [6], [7]]. However, since around the year 2000, such studies have almost disappeared. There have been reports that transverse colon interposition had poor postoperative outcomes and no benefit [8]. There were reports that ileocolon interposition after total gastrectomy had the advantages of preventing postoperative reflux esophagitis and providing functional replacement of the stomach as a reservoir for ingested food, but these were only in the era of open surgery [9,10]. We did not find poor postoperative results with ileocecal reconstruction. Ileocolic reconstruction might have become obsolete because the procedure was complicated and difficult to perform laparoscopically at that time. With the development of laparoscopic techniques and improved surgical safety, ileocolic reconstruction has been reconsidered as a reconstruction method.

We anticipate that the implementation of this reconstruction method will enhance the quality of life of patients after total gastrectomy, particularly in terms of minimizing esophageal reflux and facilitating oral ingestion. However, further validation is required. The ileocecal valve is expected to prevent esophageal reflux. Furthermore, the elevated cecum and colon may serve as reservoirs for oral intake, instead of the resected stomach. This expectation is based on the experience with ileocolic reconstruction after subtotal esophagectomy for esophageal cancer when the stomach cannot be used. This ileocolic interposition was performed as esophageal reconstruction after distal esophagectomy for esophagogastric junction cancer, which has already been described in a case report [11]. We have adapted this technique to laparoscopic total gastrectomy.

However, this reconstruction method has several disadvantages. Surgical procedures are more complicated and time-consuming than Roux-en-Y reconstruction, extending the operative time by approximately 30 min. There is also a concern that the risk of anastomotic leakage will increase owing to the presence of three anastomoses. However, in the current era, the anastomotic leakage rate for each of these remains low, at only a few percent, which is not a significant disadvantage [[12], [13], [14]]. This reconstruction may increase the risk of ileus. Following surgery for esophageal cancer, adenocarcinoma may develop in the interposition colon, albeit at a low frequency [15]. Periodic postoperative endoscopic examinations are necessary because of the possibility of metachronous elevated colon cancer.

## 4 Conclusion

We designed and performed a unique laparoscopic reconstruction using a pedicled ileocolic interposition after total gastrectomy. If both safety and functionality can be achieved, the method described in this report could become an alternative reconstruction method after laparoscopic total gastrectomy.

## Registration of research studies

1. Name of the registry

UMIN.

2. Unique identifying number or registration ID

R000063611.

3. Hyperlink to your specific registration (must be publicly accessible and will be checked):https://center6.umin.ac.jp/cgi-bin/ctr/ctr_menu_form.cgi?recptno=R000063611

## Guarantor

The Guarantor is Takeshi Ono.

## CRediT authorship contribution statement

Hisashi Fujiwara provided the authors with the idea of this study. Takeshi Ono actually performed the surgery and prepared the manuscript. Yuichiro Hirata, Koji kato, Masashi Nagata and Satoru Higa selected the patients and treated them. All authors have approved the submitted version of the manuscript and agreed to be accountable for any part of the work.

## Consent for publication

Written informed consent was obtained from all patients for publication and accompanying images. A copy of the written consent is available for review by the Editor-in-Chief of this journal upon request.

## Ethical approval

Ethical approval for this case report (approval number: 23–21) was provided by the Ethical Committee of Okinawa Kyodo Hospital, Okinawa, Japan on 25 December 2023.

## Funding

This research did not receive any specific grants from funding agencies in the public, commercial, or not-for-profit sectors.

## Declaration of competing interest

There are no conflicts of interest to declare.
